# Association between older adults’ socioeconomic status and their healthcare experiences, preferences, and attitudes towards deprescribing: a cross-sectional study in 14 countries

**DOI:** 10.1186/s13690-025-01700-6

**Published:** 2025-10-06

**Authors:** Renata Vidonscky Lüthold, Esther Kleijer-Werkman, Katharina Tabea Jungo, Zsofia Rozsnyai, Limor Adler, Radost Assenova, Eloísa Rogero-Blanco, Markus Bleckwenn, Thomas Frese, Gilles Henrard, Aisling A. Jennings, Donata Kurpas, Vanja Lazic, Heidrun Lingner, Stina Mannheimer, Anne Centeno Neelen, Anabela Pereira, Ferdinando Petrazzuoli, Rosalinde K. E. Poortvliet, Ágnes Szélvári, Dorothea M. G. Wild, Sven Streit, Enriqueta Vallejo-Yagüe

**Affiliations:** 1 Institute of Primary Health Care (BIHAM), University of Bern, 3012 Bern, Switzerland; 2 Graduate School for Health Sciences, University of Bern, 3012 Bern, Switzerland; 3 Division of Pharmacoepidemiology and Pharmacoeconomics and Center for Healthcare Delivery Sciences, Department of Medicine, Brigham and Women’s Hospital and Harvard Medical School, Boston, MA 02120 USA; 4 Department of Family Medicine, Faculty of Medical & Health Sciences, Tel Aviv University, Tel Aviv, Israel; 5 Department of Urology and General Practice, Faculty of Medicine, Medical University of Plovdiv, Plovdiv, Bulgaria; 6 General Ricardos Health Centre, Gerencia Asistencial Atención Primaria, Servicio Madrileño de Salud, Madrid, Spain; 7 Instituto de Investigación Sanitaria Gregorio Marañón, Madrid, Spain; 8 Institute of General Practice, Faculty of Medicine, Leipzig University, Leipzig, Germany; 9 Institute of General Practice and Family Medicine, Martin Luther-University Halle-Wittenberg, Halle (Saale), ST Germany; 10 Department of General Practice, Faculty of Medicine, University of Liège, Liège, Belgium; 11 Department of General Practice, University College Cork, Cork, Ireland; 12 Division of Research Methodology, Department of Nursing, Faculty of Nursing and Midwifery, Wrocław Medical University, Wrocław, Poland; 13 Health center Zagreb – Centar, Zagreb, Zagreb, Croatia; 14 Hannover Medical School, Center for Public Health and Healthcare, Department for Medical Psychology, Hannover, Germany; 15 Institute of Health and Care Sciences, Sahlgrenska Academy, University of Gothenburg, VästraGötaland Region, Gothenburg, Sweden; 16 Center for Health Technology and Services Research (CINTESIS@RISE), Department of Education and Psychology, University of Aveiro. Campus Universitário de Santiago, 3810-193 Aveiro, Portugal; 17 Institute of Biomedical Sciences Abel Salazar, University of Porto, Rua de Jorge Viterbo Ferreira, 228, 4050-313 Porto, Portugal; 18 Sezione SNaMID Caserta, Caserta, Italy; 19 Center for Primary Health Care Research, Department of Clinical Sciences, Lund University, Malmö, Sweden; 20 Department of Public Health and Primary Care, Leiden University Medical Center, Leiden, The Netherlands; 21 LUMC Center for Medicine for Older People, Leiden University Medical Center, Leiden, The Netherlands; 22 Department of Family Medicine, Semmelweis University, Budapest, Hungary; 23 Institute of Family Medicine and General Practice, University Hospital Bonn, Bonn University, Bonn, Germany

**Keywords:** Deprescribing, Socioeconomic status, Polypharmacy, Patient care, Older adults

## Abstract

**Background:**

Socioeconomic status (SES) can influence health outcomes. Both SES and older age are associated with polypharmacy, health literacy, and quality of care. Understanding how SES influences healthcare experiences of older adults with polypharmacy can serve to inform future interventions aiming at optimising patient care. Therefore, we investigated the association between older patients’ SES and their i) attitudes towards deprescribing, ii) satisfaction with medications, iii) self-rated health, iv) health literacy, and v) trust in their general practitioner (GP).

**Methods:**

In this cross-sectional study, older patients with polypharmacy from 14 countries completed a survey on their attitudes towards deprescribing, healthcare experiences, and sociodemographic characteristics. We compared patients’ responses across high (reference), middle, and low SES groups (defined by education and financial status), and performed multilevel logistic regressions adjusted for clustering at the country level to assess the association between patients’ SES and the outcomes.

**Results:**

Among 1,320 older adults, compared to those with high SES, patients with low SES were more likely to want a medication deprescribed (OR_lowSES_ 1.76, 95%CI 1.20-2.57). Those with medium SES were less likely to trust their GP (OR_mediumSES_ 0.70, 95%CI 0.52-0.94). Both low and medium SES groups were less likely to be satisfied with their current medications (OR_lowSES_ 0.45, 95%CI 0.29-0.71; OR_mediumSES_ 0.63, 95%CI 0.44-0.92), less likely to report good health (OR_lowSES_ 0.22, 95%CI 0.14-0.34; OR_mediumSES_ 0.49, 95%CI 0.37-0.65), and had lower health literacy (OR_lowSES_ 0.10, 95%CI 0.07-0.16; OR_mediumSES_ 0.31, 95%CI 0.24- 0.41).

**Conclusion:**

Older adults with lower SES expressed greater interest in deprescribing, lower satisfaction with medications, lower self-rated health, and lower health literacy. Our findings suggest key aspects to consider when optimising care of older adults with low SES.

**Supplementary Information:**

The online version contains supplementary material available at 10.1186/s13690-025-01700-6.

**Table:** 

**Text box 1. Contributions to the literature**
• Socioeconomic status (SES) may influence older adults’ attitudes towards medication, healthcare experiences and preferences.
• A multi-country study mitigates country-related differences that may influence the impact of SES on healthcare.
• Understanding how SES influences healthcare experiences and preferences of older adults with polypharmacy may inform future interventions aiming at improving care equity while optimising patient care.
• Older adults with lower SES expressed greater interest in deprescribing, lower satisfaction with medications, lower self-rated health, and lower health literacy.
• SES should be considered in clinical practice and when designing tailored interventions for the older population.

## Background

Promoting healthy ageing and health equity is essential to addressing the growing challenges in the health care of older adults [35]. Health promotion strategies are most effective when tailored to patients’ preferences and behaviours [4]. Healthcare and medication-related behaviours of older adults are shaped by a complex interplay of cognitive, social, and structural factors, including health literacy [22], trust in providers [38], and socioeconomic disparities [6, 16]. Low socioeconomic status (SES) has been associated with poorer health outcomes, including lower life expectancy, lower quality of life, limited health literacy, insufficient knowledge about medications, poor medication adherence, and higher morbidity and mortality [6, 16]. Moreover, older adults with low SES are more likely to experience polypharmacy (regular use of ≥5 medications [28]) [13, 33], which often reflects a higher prevalence of comorbidities and less access to preventive medications among these patients [6, 14, 16, 17, 19]. Such SES-related disparities place older adults with polypharmacy and low SES at potentially increased risk for medication-related harm and greater healthcare inequities. However, the relationship between older adults’ SES and their attitudes towards medication use remains little explored.

One of the strategies to optimise medication use is deprescribing (the process of stopping or reducing inappropriate medications) [41]. Patients’ willingness to deprescribe was inconsistently associated with educational level and financial aspects [51]. Deprescribing behaviours might be influenced not only by cognitive factors (e.g., medication literacy [12]) but also by environmental and social reinforcements (e.g., provider communication [46, 49], and perceived cost benefits [15]). Patient-provider relationship and trust in the physician may influence shared decision-making and impact patients’ attitudes towards deprescribing [18, 25, 43, 46, 49]. Patients with lower SES tend to have less engagement in medical consultations [1, 32], which in turn could impact their involvement in shared decision-making and their satisfaction with patient care [40, 48]. However, it is unclear how older adults’ SES influences their trust in the physician. Furthermore, ageism in healthcare, including implicit biases that undervalue older adults'preferences, autonomy and capacities for shared decision-making [39], may further exacerbate disparities in medication use and patient care. Lower health literacy observed in persons with low SES may exacerbate older adults’ struggles to understand their medication regimens and participation in informed healthcare decisions [6, 40]. Nevertheless, the interplay between older adults’ SES and health literacy, attitudes toward deprescribing, trust in the physician, satisfaction with medications and health status has not been comprehensively examined across diverse international contexts. Understanding how SES influences older adults’ healthcare experiences and preferences will shed light on future interventions focusing on improving care equity while optimising patient care.

SES is not only a demographic variable but also a determinant of patient experiences and behaviour, potentially influencing medication management and healthcare interactions. However, there is little evidence focusing on the perceptions of older adults with polypharmacy and exploring how their SES affects the care they receive and their preferences [3]. The relationship between SES and healthcare outcomes is complex and multidimensional. The impact of SES on healthcare might not only be influenced by individual experiences, but also by differences in healthcare settings, systems, and cultural context [16, 21]. Therefore, a multi-country study, assessing SES and healthcare outcomes in a unified manner, would mitigate country-related outcome differences when exploring the potential impacts of SES on healthcare experiences and perspectives of older adults.

To be able to address health inequities and support older adults with polypharmacy and low SES when optimising medications, we need to better understand SES-related disparities associated with patient attitudes towards medication use, as well as their experiences and preferences in patient care. Therefore, our study explores how SES influences key patient-reported outcomes relevant to medication management. More specifically, this study aims to investigate the association between older patients’ SES and their i) attitudes towards deprescribing, ii) satisfaction with medications, iii) self-rated health, iv) health literacy, and v) trust in their general practitioner (GP). 

## Methods

### Study design and data source

This cross-sectional survey study is a post-hoc analysis of the “Understanding older patients’ willingness to have medications deprescribed in primary care” study (host study) [26]. The host study was conducted in primary care settings at 17 sites in 14 countries. In each country, national coordinators targeted the recruitment of 100 patients per country through GPs. Each GP was asked to recruit 10 patients to respond to an anonymous survey. The questionnaire included questions on socio-demographic characteristics, trust in their GP, and attitudes towards deprescribing. It was translated and cross-culturally adapted for each participating country by the respective national coordinator(s), and it could be completed on paper or online using the REDCap^®^ survey function [36]. Further details on the primary study design were published elsewhere [26].

### Study population

Inclusion criteria in the primary study were being ≥65 years old and having polypharmacy (regular use of ≥5 medications). Exclusion criteria were inability to give informed consent and/or residency outside of the participating countries (Belgium, Bulgaria, Croatia, Germany, Hungary, Ireland, Israel, Italy, Netherlands, Poland, Portugal, Spain, Sweden, and Switzerland) [26]. Additional exclusion criteria for this secondary analysis were absence of response to the questions *‘How do you make ends meet financially?’* and/or the question *‘What is your highest completed education?’*. Moreover, patients with missing outcome data were excluded from the analysis on an outcome basis (see *Outcome* paragraph below).

### Exposure

The study exposure was SES, classified as low SES, middle SES, and high SES. Education level and financial status are commonly used as a proxy for SES [24], therefore SES was defined by the combination of the highest level of education and the financial situation, as indicated in Table 1. To assess the highest level of education, we used the question *‘What is your highest completed education?’*, categorised as 1) none, 2) primary education, 3) secondary school (high school or vocational training), 4) third level education (university or equivalent training). To assess the financial situation, we used the question: *‘How do you make ends meet financially?’*, categorised as: 1) with great difficulty, 2) with some difficulty, 3) quite easily, 4) without any problems (adapted from the EU Statistics on Income and Living Conditions (EU-SILC) survey [11].

**Table 1:** Classification of socioeconomic status (SES) groups

Highest completed education^a^						
None				Primary education	Secondaryschool	Third level education
**Financial situation**^2^			**Low**		**High**	
	With great difficulty	**Low**	Low SES		Middle SES	
	With some difficulty					
	Quite easily	**High**	Middle SES		High SES	

^a﻿^To assess the highest level of education, we used the question *‘What is your highest completed education?’ *^2^To assess the financial situation, we used the question: *‘How do you make ends meet financially?’*

### Outcomes

The study has five outcomes: 1) interest in having medications deprescribed, 2) satisfaction with medications, 3) self-rated health, 4) health literacy, and 5) trust in the GP. These were defined as follows:

*Interest in having medications deprescribed* was assessed by the question *‘Thinking about your current medications, are there any that you would like to stop or reduce?’*. Patients who responded *‘yes*’ to this question were considered as wanting to have a medication deprescribed, patients who responded *‘no’* were classified as not wanting to have medication deprescribed.

*Satisfaction with medications* was assessed by the 5-point Likert scale question *‘Overall, I am satisfied with my current medications’*. *‘Strongly agree’* and ‘*agree’* were considered as satisfied with medications, while *‘I do not know’, ‘disagree’,* and *‘strongly disagree’* were classified as not satisfied [42].

*Self-rated health* was assessed by the question *‘In general, how would you describe your health today?*’. Responses *‘excellent’*, *‘very good’, and ‘good’* were considered as good self-rated health, while *‘average’*, and *‘poor’* were considered not good [27, 29].

*Health literacy* was assessed by the question *‘How confident are you filling out medical forms by yourself?*’. Responses *‘not at all’*, *‘a little bit’*, or ‘*somewhat’* were considered as low health literacy, while ‘*quite a bit’* and ‘*extremely’* were considered as high health literacy [7].

*Trust in the GP* was classified as *‘high trust’* and *‘low trust’*, assessed using the abbreviate Wake Forest Trust in Physician Scale [10]. This scale has five 5-point Likert scale questions to assess patient trust in the physician, and an overall score from 5 to 25, with higher values indicating higher trust. The patient trust score was dichotomised into high trust (score ≥ to the median) and low trust (score < the median).

### Covariates

Other variables included in the study were: patient gender, duration of GP-patient relationship (in 20-year bins), born in the country they live in, number of regular medications, and GP gender.

### Statistical analysis

We used descriptive statistics to depict patient characteristics. Continuous variables were presented as median and interquartile range (IQR), and categorical variables as numbers and percentages. We used chi-square tests, Fisher’s exact test, and Mann-Whitney U test to compare patient characteristics and attitudes towards deprescribing across SES groups. Subsequently, to investigate the binary outcomes (i.e., interest in deprescribing, satisfaction with medications, self-rated health, trust in the GP, and health literacy) we performed multilevel multivariate logistic regressions, accounting for cluster effects at the country level. We calculated the intra-cluster correlation (ICC) in the regression models to explore the country variability in our model [31]. The regressions were done crude and adjusted for potential confounders. We used a hypothesis-driven approach to select the potential confounders, and these included: patient’s gender, duration of GP-patient relationship, patients’ birth country (*‘Were you born in the country you currently live in?’* (yes/no)), and GP’s gender. We used complete case analysis in an outcome-basis, and to deal with missing data in the covariates. We used Stata 16.1 (StataCorp, College Station, TX, USA) to perform the analysis. A two-sided p-value of 0.05 was considered statistically significant. 

## Results

**Fig 1:**
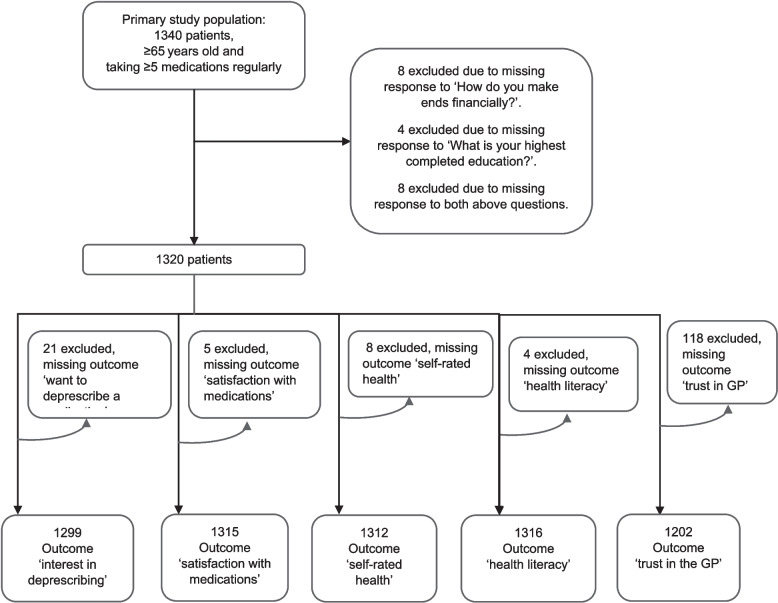
Flowchart of the recruitment of patients

There were 1,340 older adults who completed the questionnaire of the primary study, of which 1,320 complete cases (i.e., had data on SES) were included in this secondary analysis study (Fig. 1 and Figure S1). Most of the patients reported being female (*n*=725, 55%), having completed secondary school education (*n*=576, 44%), making ends meet quite easily (*n*=451, 34%) or with some difficulty (*n*=446, 34%), and having a good overall health (*n*=783, 59%) (Table 2). Patients were taking a median of 7 medications (IQR=3) and most were satisfied with their current medications (*n*=1,074, 81%) (Table 2). Among the participants, 582 (44%) would like to have at least one medication deprescribed and 1,073 (81%) would be willing to have a medication deprescribed if their doctor recommended it. Classifying the patients into SES groups, 638 (48%) had high SES, 465 (35%) had medium SES, and 217 (16%) had low SES (Table S1).

**Table 2:** Patient characteristics by socioeconomic status (SES) (*n*=1,320)

	**Overall**	**High** **SES** (ref.)	**Middle** **SES**	***p*-value**	**Low** **SES**	***p*-value**
**Patient characteristics**	*n* = 1320	*n* = 638	*n* = 465		*n* = 217	
**Gender **^**a**^, n (%)				**0.000**		**0.019**
Female	725 (55%)	311 (49%)	288 (62%)		126 (58%)	
Male	593 (45%)	326 (51%)	176 (38%)		91 (42%)	
**Born in the country of residence**, n (%)				0.386		**0.002**
Yes (*versus* no)	1207 (91%)	574 (90%)	424 (91%)		209 (96%)	
**Living area**, n (%)				**0.001**		**0.000**
Urban area	691 (52%)	299 (47%)	268 (58%)		124 (57%)	
Suburban area	372 (28%)	222 (35%)	117 (25%)		33 (15%)	
Rural	255 (19%)	115 (18%)	80 (17%)		60 (28%)	
**Medication preparation**, n (%)				0.104		**0.000**
Self-prepare and take medication(s)	1148 (87%)	585 (92%)	415 (89%)		148 (68%)	
Receive support managing medication(s)	166 (13%)	49 (8%)	49 (11%)		68 (31%)	
**Number of regular medications**, median (IQR)	6 (5-8)	6 (5-8)	6 (5-8)	**0.010**	7 (5-8)	**0.002**
**Willingness to deprescribe (rPATD)** ^b^				**0.028**		0.158
Yes	1,073 (81%)	535 (84%)	366 (79%)		172 (79%)	
No	235 (18%)	98 (15%)	95 (20%)		42 (19%)	
**GP gender** ^c,d^, n (%)				0.171		0.060
Female	686 (52%)	309 (48%)	249 (54%)		128 (59%)	
Male	543 (41%)	277 (43%)	183 (40%)		83 (38%)	
Other	15 (1%)	10 (2%)	4 (1%)		1 (1%)	
**Duration GP relationship** ^c,e^, n (%)				0.161		0.304
<20 years	980 (77%)	460 (72%)	353 (76%)		167 (77%)	
20+ years	290 (23%)	151 (24%)	94 (20%)		45 (21%)	
**GP practice location** ^c^, n (%)				**0.000**		**0.000**
Urban area	762 (58%)	330 (52%)	289 (62%)		143 (66%)	
Suburban area	292 (22%)	183 (29%)	85 (18%)		24 (11%)	
Rural	153 (12%)	61 (10%)	51 (11%)		41 (19%)	

Abbreviations: *SES* Socioeconomic status, *N* Number, Sample size, *IQR* Interquartile range, *GP* General practitioner

*p*-values calculated with chi-square test or Fisher’s exact test or Mann-Whitney U test, reference category: High SES

The missing data was ≤1% for all variables, except for: ‘Number of medications’ (3%); ‘Duration of the patient-GP relationship’ (4%); ‘GP gender’ and ‘source of knowledge about medications’ (6%); ‘GP's practice location’ (9%); ‘Want to stop or reduce a medication’ (2%). Percentages are calculated considering missingness

^a^ No patient chose the option *‘other’* to the question ‘*What is your gender?*’

^b^ From the revised patient attitudes towards deprescribing questionnaire [42]

^c^ Only shown for patients who responded *‘yes’* to the question ‘*Do you have your own GP/family doctor (definition: when you have a health problem, you usually consult the same family doctor, except in emergencies)?*’ (*n*=1,276)

^d^ 8 GPs classified as ‘*other*’ were from the Netherlands, where the patients in our sample did not have a unique fixed GP

^e^ Categories of patient-GP relationship were: <10, 10-19, 20-29, 30+ years, dichotomised into >/< 20 years

Across SES groups, the proportion of males decreased with lower SES categories (51%, 38%, 42% in high, medium, low SES, respectively), the percentage of persons born in the country of residence increased (90%, 91%, 96%, in high, medium, low SES, respectively), and the willingness to deprescribe (rPATD) decreased (84%, 79%, 79%, high, medium, low, respectively). Different distribution of living area (urban, suburban, rural) was observed across groups, with a similar pattern of change than the GP practice location (Table 2).

**Fig 2:**
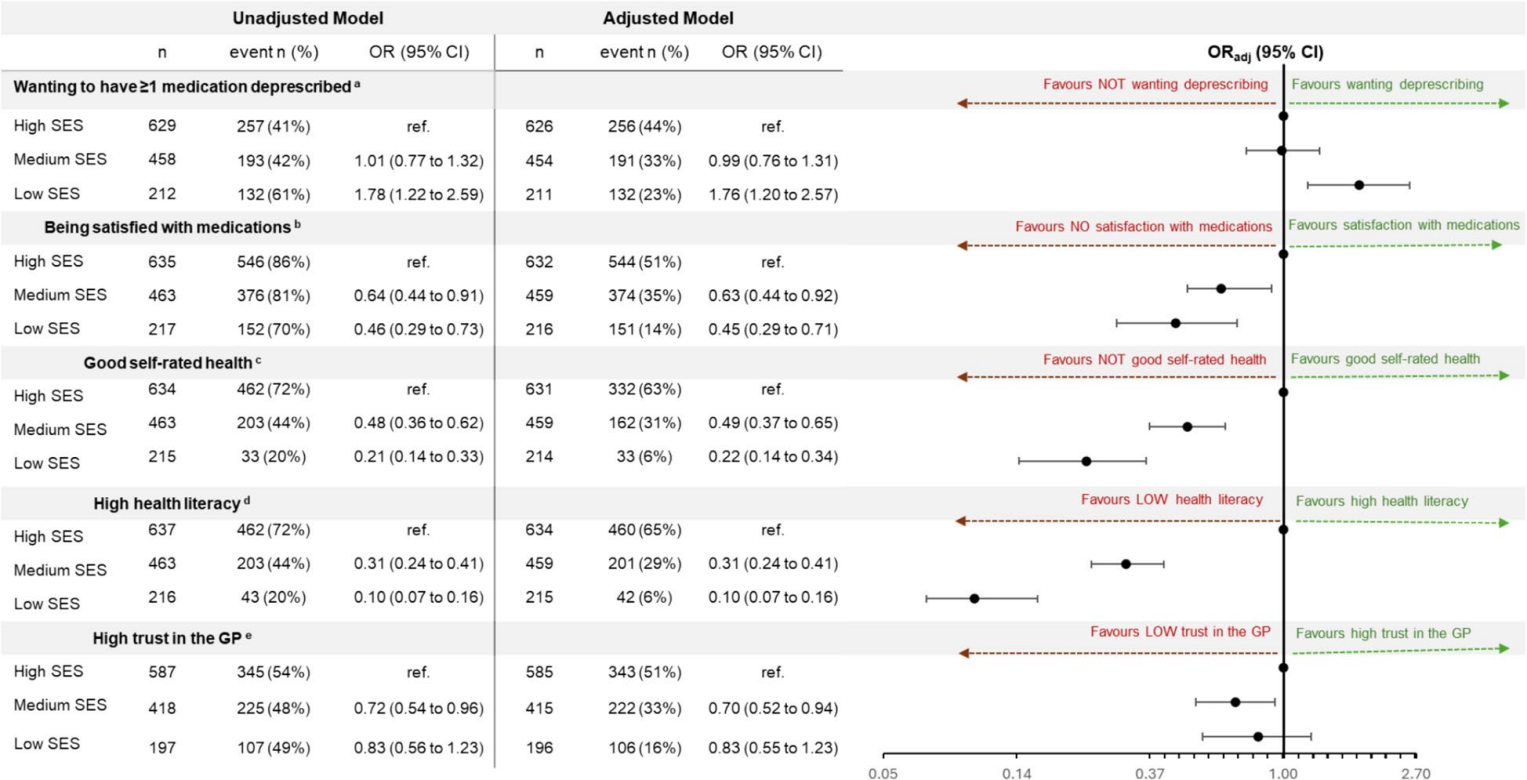
Association between socioeconomic status (SES) and patient attitudes towards deprescribing, satisfaction with medications, self-rated health, health literacy, and trust in the General Practitioner (GP)

Compared with older adults with high SES, those with low SES had higher odds of wanting to have a medication deprescribed compared to those with high SES (OR_lowSES_ 1.76, 95%CI 1.20-2.57) (Fig. 2). Older adults with lower SES had lower odds of reporting being satisfied with their current medications (OR_lowSES_ 0.45, 95%CI 0.29-0.71; OR_mediumSES_ 0.63, 95%CI 0.44-0.92), lower odds of reporting excellent/very good/good health (OR_lowSES_ 0.22, 95%CI 0.14-0.34; OR_mediumSES_ 0.49, 95%CI 0.37-0.65) and lower odds of reporting high health literacy (OR_lowSES_ 0.10, 95%CI 0.07-0.16; OR_mediumSES_ 0.31, 95%CI 0.24- 0.41) (Fig. 2) compared to older adults with high SES. In addition, patients with medium SES had lower odds of reporting high trust in their GP compared with those with high SES (OR_mediumSES_ 0.70, 95%CI 0.52-0.94) (Figure 2).

## Discussion

In this study involving 1,320 older adults with polypharmacy from 14 countries, the SES of older adults was associated with attitudes towards deprescribing, satisfaction with medications, self-rated health, health literacy, and trust in the GP. Compared to older adults with polypharmacy with high SES, those with low SES were more likely to want a medication deprescribed. Additionally, older adults with low or medium SES were less satisfied with their medications, more likely to report lower self-rated health and low health literacy vs those with high SES. Our findings suggest the importance of considering SES when addressing healthy ageing and medication optimisation for older adults with polypharmacy.

In the literature, inconsistent results have been reported on the association between educational levels and willingness to deprescribe [34, 42, 51]. While a survey study in Malaysia [20] reported patients with lower educational levels to be more willing to deprescribe, another survey study in Switzerland found higher education to be associated with higher willingness [45], and other studies found no association [2, 37]. Of note, while our outcome was based on a question asking whether patients *would like to* have any of their own medications deprescribed; other studies assessed patients’ *willingness* to deprescribe *if their physician said it was possible* (i.e., using the rPATD questionnaire [2, 20, 37, 45]. Different ways to assess patient attitudes towards deprescribing might influence such associations. For example, when the doctor is mentioned in the question, patients may tend to agree more with deprescribing - although this still requires confirmatory research [8]. The novelty of our study focusing on SES and older adults’ *wish* to deprescribe specific medications should be further explored in studies with different designs and settings.

The higher interest in deprescribing among older adults with lower SES in our study may be related to the higher economic vulnerability in this group. However, given the different healthcare systems across the study countries, the economic burden of medications may geographically differ. It may also be related to their medication adherence, in line with ecological models [5]. Furthermore, the increased interest in deprescribing in the lower SES group aligns with their reported lower satisfaction with medications. This is supported by evidence that low SES patients may be less likely to challenge healthcare professionals [40, 48], which may lead to discordances between prescribed treatments and patient expectations, potentially resulting in discontent with their medications. These findings suggest that implementing SES-sensitive medication-related interventions and communication could improve patient satisfaction.

Former evidence on the association between SES and trust in the physician is limited and conflicting [23, 32, 44], and does not focus on older adults. Individuals with low SES experience more health barriers [30, 47], which could influence trust in the system. Similarly, the lower likelihood of questioning medical advice and lower involvement in medical consultations described in persons with low SES [1, 9, 32, 40, 44, 48] suggest behaviours that could be linked to the trust these patients have in their prescribing doctor. In our study, there is a tendency of reduced trust when reducing SES group, but it is not a clear pattern. We observed reduced trust in the GP in the medium SES group, but not significant reduction in the low SES. It is possible that the high trust in the GP among the overall of study participants masked a potential association between low SES and trust in the GP.

Lower access to health information have been described in persons with low SES [30, 47]. This is in line with the lower health literacy in older adults with lower SES observed in our study, which may reflect higher burdens in understanding and adhering to their medication regimens. A good understanding of medications is necessary for informed decision-making and to ensure that patients take their medications as prescribed. Patients with limited health literacy may be unaware of the benefits of the medication and of the reasons for taking it [40, 48], which may also reflect the higher interest in deprescribing among older adults with low SES. This is supported by the Hierarchical Model for Medication Adherence, which suggests that adequate health literacy is crucial for medication adherence [50]. Moreover, low SES has been associated with a higher number of comorbidities and worsened quality of life [6, 16], in line with our findings of lower self-rated health in the low SES group.

Following the observed lower trust in the GP, lower health literacy, and worse self-rated health among the lower SES groups, future studies may explore whether improving health literacy in low SES populations might enhance patient-provider communication and relationship, which in turn could reflect in patient outcomes (e.g., self-rated health and satisfaction with medications). While this rationale remains speculative, it points to a potentially important pathway that could be targeted in interventions aimed at reducing SES-related disparities in care and outcomes.

In conclusion, we found that compared to older adults with polypharmacy with higher SES, those with lower SES had lower satisfaction with medications and greater interest to deprescribe, lower health literacy, and lower self-rated health. Thus, SES may play an important role in the healthcare experiences of older adults with polypharmacy, and this should be considered in clinical practice and when designing tailored interventions for the older population. For instance, targeted educational interventions to promote medication literacy and health education in lower SES groups could in turn empower patients to make informed decisions and actively participate in the medication optimisation process.

### Strengths and limitations

Our study is strengthened by its wide scope, addressing several personal factors, as well as its international design, involving 14 different countries. Another strength is its novel aspect of exploring the association of SES, deprescribing, and healthcare experiences focusing on older adults, a vulnerable population that can benefit from multiple health promotion initiatives. This study has also limitations. The overall high health literacy, high SES, good self-rated health, high trust in the GP, and low representativeness of immigrants in our study population limits the overall generalisability of our findings and may have potentially led to an underestimation of the association between SES and the study outcomes. Considering that we found associations despite the overall high SES, it is possible that by involving populations under-represented in our study (e.g., with lower SES), the associations found in our study would be stronger.

Regarding the SES classification, despite existing different definitions of SES, our approach including education level and financial status encompasses commonly used proxies for SES [24]. And while the question assessing patients’ financial status could have been influenced by individual perceptions and potential biases, the EU-SILC methodology is widely recognised and used in research.

In the study, GPs were instructed to recruit patients consecutively, but we cannot rule out selection bias. If GPs selected patients with whom they have a good relationship, this could have resulted in higher than expected reported trust in their GP. Although patient-provider relationship also may have influenced study participation, having GPs recruit patients was a feasible approach considering that they had access to patients’ health information and could recruit eligible patients during consultations. And due to the data collection strategy, we were unable to track response rates. In addition, we defined polypharmacy as the regular use of 5 or more medications, without considering the active pharmaceutical ingredients. While considering the active ingredients could be more informative, using the medications was a pragmatic and widely applied approach in studies on polypharmacy [28]. While our questionnaire assessed a large number of variables, unmeasured confounders cannot be ruled out, and mediators and effects modifiers were not investigated. We acknowledge the different male/female ratio across SES groups, and the lack of representation of gender minorities (with zero participants self-identifying as other than female or male, despite given choice). The gender perspective of this study remains to be explored. Additionally, we acknowledge differences in the living area (urban, suburban, rural) across the SES groups. While initially this could indicate differences in geographical access to point of care, the very similar pattern in the GP practice location (urban, suburban, rural) across groups, suggest a likely similar distance to the GP. Lastly, we did not collect the exact age of the participants, preventing us from adjusting the analysis for age. However, the study’s focus on older adults ensures an age-restricted and relatively homogeneous population, which minimizes the impact of age variability on our findings.

## Conclusion

The higher interest in deprescribing, lower satisfaction with medications, lower self-rated health, and lower health literacy among older adults with lower SES suggest that SES impacts the healthcare experiences of older adults, and their perceptions and attitudes towards medication use. Therefore, SES should be considered when optimising patient care (e.g., when designing patient educational materials). By addressing SES-related disparities, healthcare providers can better support older adults in managing their medications and improving their overall healthcare experiences.

## Supplementary Information

Supplementary Material 1.

## Data Availability

The data for this study are available to other researchers on request. The data will be made available for scientific research purposes, after the proposed analysis plan has been approved by the core study team. Data and documentation will be made available through a secure file exchange platform after approval of the proposal. In addition, a data transfer agreement must be signed (which defines obligations that the data requester must adhere to regarding privacy and data handling). For data access, please contact the corresponding author.
