# Supplementary material for: Association between older adults’ socioeconomic status and their healthcare experiences, preferences, and attitudes towards deprescribing: a cross-sectional study in 14 countries
Source: Arch Public Health. 2025 Oct 6;83:237. doi: 10.1186/s13690-025-01700-6 (PMC12502375; doi:10.1186/s13690-025-01700-6)
Supplement: Supplementary file 1 — Supplementary Material 1. [file 13690_2025_1700_MOESM1_ESM.docx]

**Association between older adults’ socioeconomic status and their healthcare experiences, preferences, and attitudes towards deprescribing: A cross-sectional study in 14 countries**

Renata Vidonscky Lüthold ^1,2^, Esther Kleijer-Werkman ^1^, Katharina Tabea Jungo ^1,3^, Zsofia Rozsnyai ^1^, Limor Adler ^4^, Radost Assenova ^5^, Eloísa Rogero-Blanco ^6,7^, Markus Bleckwenn ^8^, Thomas Frese ^9^, Gilles Henrard ^10^, Aisling A. Jennings ^11^, Donata Kurpas ^12^, Vanja Lazic ^13^, Heidrun Lingner ^14^, Stina Mannheimer ^15^, Anne Centeno Neelen ^1^, Anabela Pereira ^16,17^, Ferdinando Petrazzuoli ^18,19^, Rosalinde K. E. Poortvliet ^20,21^, Ágnes Szélvári ^22^, Dorothea M. G. Wild ^23^, Sven Streit ^1^, Enriqueta Vallejo-Yagüe^1,^**^^**

^1^ Institute of Primary Health Care (BIHAM), University of Bern, 3012 Bern, Switzerland

^2^ Graduate School for Health Sciences, University of Bern, 3012 Bern, Switzerland

^3^ Division of Pharmacoepidemiology and Pharmacoeconomics and Center for Healthcare Delivery Sciences, Department of Medicine, Brigham and Women's Hospital and Harvard Medical School, 02120 Boston, MA, USA

^4^ Department of Family Medicine, Faculty of Medical & Health Sciences, Tel Aviv University, Tel Aviv, Israel

^5^ Department of Urology and General Practice, Faculty of Medicine, Medical University of Plovdiv, Plovdiv, Bulgaria

^6^ General Ricardos Health Centre, Gerencia Asistencial Atención Primaria, Servicio Madrileño de Salud, Madrid, Spain

^7^ Instituto de Investigación Sanitaria Gregorio Marañón, Madrid, Spain

^8^ Institute of General Practice, Faculty of Medicine, Leipzig University, Leipzig, Germany

^9^ Institute of General Practice and Family Medicine, Martin Luther-University Halle-Wittenberg, Halle (Saale), ST, Germany

^10^ Department of General Practice, Faculty of Medicine, University of Liège, Liège, Belgium

^11^ Department of General Practice, University College Cork, Cork, Ireland

^12^ Division of Research Methodology, Department of Nursing, Faculty of Nursing and Midwifery, Wrocław Medical University, Wrocław, Poland

^13^ Health center Zagreb – Centar, Zagreb, Croatia

^14^ Hannover Medical School, Center for Public Health and Healthcare, Department for Medical Psychology, Hannover, Germany

^15^ Institute of Health and Care Sciences, Sahlgrenska Academy, University of Gothenburg, Gothenburg, Västra Götaland Region, Sweden

^16^ Center for Health Technology and Services Research (CINTESIS@RISE), Department of Education and Psychology, University of Aveiro. Campus Universitário de Santiago, 3810-193, Aveiro, Portugal

^17^ Institute of Biomedical Sciences Abel Salazar, University of Porto, Rua de Jorge Viterbo Ferreira, 228, 4050-313, Porto, Portugal

^18^ Sezione SNaMID Caserta, Caserta, Italy

^19^ Center for Primary Health Care Research, Department of Clinical Sciences, Lund University, Malmö, Sweden

^20^ Department of Public Health and Primary Care, Leiden University Medical Center, Leiden, The Netherlands

^21^ LUMC Center for Medicine for Older People, Leiden University Medical Center, Leiden, The Netherlands

^22^ Department of Family Medicine, Semmelweis University, Budapest, Hungary

^23^ Institute of Family Medicine and General Practice, University Hospital Bonn, Bonn University, Bonn, Germany

**^^Corresponding author:** [enriqueta.vallejo@unibe.ch](mailto:enriqueta.vallejo@unibe.ch)

### **Supplementary Files**

### **Figure S1. Recruitment flow chart.**

## **Enrolment**

## Patients screened by general practitioners and handed a questionnaire (n=1,423)

## Patients excluded:

## Did not give informed consent: n=19

- Did not meet eligibility criteria: n=58

## < 65 years old: n=25

## < 5 regular medications: n=33

- Responded to less than 5 questions: n=6
- No financial information: n=16
- No educational information: n=4

## Participants completing the questionnaire by country (alphabetical order) (n=1,320)

## Belgium: n=100

## Bulgaria: n=93

## Croatia: n=27

## Germany: n=90

## Hungary: n=98

## Ireland: n=28

## Israel: n=64

## Italy: n=92

## Netherlands: n=224

## Poland: n=108

## Portugal: n=91

## Spain: n=103

## Sweden: n=101

## Switzerland: n=101

## **Analysed**

**Table S1.** Classification of the patients according to socioeconomic status (SES) (n=1,320)

|  | | | **HIGHEST COMPLETED EDUCATION ^1^** | | | |
| --- | --- | --- | --- | --- | --- | --- |
|  |  |  | None | Primary education | Secondary school | Third level education |
| **Financial situation** ^2^ |  |  | **Low** | | **High** | |
|  | With great difficulty | **Low** | Low SES  217 (16%) | | Middle SES  313 (24%) | |
|  | With some difficulty |  |  |  |  |  |
|  | Quite easily | **High** | Middle SES  152 (12%) | | High SES  638 (48%) | |
|  | Without any problems |  |  |  |  |  |

^1^ To assess the highest level of education, we used the question *‘What is your highest completed education?’*

^2^ To assess the financial situation, we used the question: *‘How do you make ends financially?’*.
